# P2X_3_ Receptor in Primary Afferent Neurons Mediates the Relief of Visceral Hypersensitivity by Electroacupuncture in an Irritable Bowel Syndrome Rat Model

**DOI:** 10.1155/2020/8186106

**Published:** 2020-09-16

**Authors:** Fang Zhang, Zhe Ma, Zhijun Weng, Min Zhao, Handan Zheng, Luyi Wu, Yuan Lu, Chunhui Bao, Yanan Liu, Huirong Liu, Huangan Wu

**Affiliations:** ^1^Shanghai Research Institute of Acupuncture and Meridian, Shanghai University of Traditional Chinese Medicine, Shanghai, China; ^2^Shanghai University of Traditional Chinese Medicine, Shanghai, China

## Abstract

**Background:**

Electroacupuncture (EA) has been confirmed effectiveness in the treatment of irritable bowel syndrome (IBS), and P2X_3_ receptors in the peripheral and central neurons participate in the acupuncture-mediated relief of the visceral pain in IBS.

**Objective:**

To reveal the neurobiological mechanism that P2X_3_ receptor of colonic primary sensory neurons in the dorsal root ganglia of the lumbosacral segment is involved in the alleviation of visceral hypersensitivity by EA in an IBS rat model.

**Methods:**

The IBS chronic visceral pain rat model was established according to the method of Al-Chaer et al. EA at the bilateral He-Mu points, including ST25 and ST37, was conducted for intervention. The behavioral studies, histopathology of colon, electrophysiology, immunofluorescence histochemistry, and real-time polymerase chain reaction assays were used to observe the role of P2X_3_ receptor in the colon and related DRG in relieving visceral hypersensitivity by EA.

**Results:**

EA significantly reduced the behavior scores of the IBS rats under different levels (20, 40, 60, 80 mmHg) of colorectal distention stimulation and downregulated the expression levels of P2X_3_ receptor protein and mRNA in colon and related DRG of the IBS rats. EA also regulated the electrical properties of the membranes, including the resting membrane potential, rheobase, and action potential of colon-associated DRG neurons in the IBS rats.

**Conclusion:**

EA can regulate the P2X_3_ receptor protein and mRNA expression levels in the colon and related DRG of IBS rats with visceral pain and then regulate the excitatory properties of DRG neurons.

## 1. Introduction

Irritable bowel syndrome is a chronic functional bowel disorder [1, 2]. Visceral hypersensitivity is one of the main pathophysiological mechanisms of IBS [3, 4]. Numerous studies have shown that P2X receptors, mainly the P2X_3_ and P2X_2/3_ receptor subtypes, are involved in visceral pain signaling [5, 6]. Burnstock et al. [7, 8] confirmed that P2X_3_, P2X_2/3_, and other receptors can transmit nociceptive information to the pain center and raised the hypothesis that “the essence underlying the effects of traditional Chinese acupuncture is the purinergic signal” [9], which provides a new idea for research on the mechanism of acupuncture.

Acupuncture can play a role in relieving visceral pain by acting on both gastrointestinal motility [10] and paresthesia. Our previous clinical and animal studies have also confirmed the effectiveness of acupuncture in the treatment of IBS [11–13] and initially revealed that the P2X_2_, P2X_3_, and P2Y_1_ receptors in the peripheral neurons of the colon and in central neurons participate in the acupuncture-mediated relief of the visceral pain in IBS. Especially, P2X_3_ receptors play an important role in mediating the occurrence and maintenance of pain in neurons of the intestinal myenteric plexus, dorsal root ganglia (DRG), spinal dorsal horn, prefrontal cortex, and anterior cingulate cortex in a rat model of IBS with visceral hypersensitivity [14], and acupuncture can achieve visceral pain relief through purinergic receptors at different levels of the brain-gut axis. We revealed the scientific basis of EA at the He-Mu points for the treatment of IBS visceral hypersensitivity from the perspective of the regulation of P2X_2_, P2X_3_, and P2Y_1_ and provided an experimental basis for the interpretation of the mechanism underlying the acupuncture effect. However, there is still no electrophysiological evidence supporting the participation of P2X_3_ receptors in the primary afferent sensory nerve of the colon in the EA-mediated inhibition of peripheral sensitization.

In this study, starting from the mechanism by which P2X_3_ receptors in the colon and colon-associated DRG are involved in the EA-mediated alleviation of IBS visceral hypersensitivity, we investigated the acupuncture-mediated regulation of the peripheral sensitization of visceral pain to provide an experimental basis for the interpretation of the mechanism by which acupuncture relieves visceral pain.

## 2. Methods

### 2.1. Animals

Eight-day-old neonatal SD (Sprague-Dawley) male rats were provided by the Animal Experimental Center of Shanghai University of Traditional Chinese Medicine (animal license number: SCXK (Shanghai) 2013-0016). The neonatal rats were housed 6 in a cage with 1 lactating rat. Lactating rats were fed with free access to food and drinking water. The housing environment was as follows: a 12 : 12 dark-light cycle, room temperature of 20 ± 2°C, and indoor humidity of 50-70%. The laboratory animal treatments were in accordance with the guidelines of the International Association for the Study of Pain (IASP).

### 2.2. Induction of Visceral Hypersensitivity

An operation procedure was prepared to establish the chronic visceral hypersensitivity rat model according to the process described by Al-Chaer et al. [15]. Rats were given colorectal dilatation (CRD) stimulation in the awake state. During the operation, the surface of a balloon was first lubricated with an appropriate amount of liquid paraffin and then slowly inserted from the anus along the physiological rectal curvature of the neonatal rats to a depth of approximately 2 cm, reaching the position of the descending colon. The balloon was inflated approximately 0.2 ml using a syringe for 1 min, followed by deflation, and the same stimulation was repeated once after 1 h. After the end of the stimulation, the balloon was removed, and the procedure was conducted once a day for 14 days.

### 2.3. Visceromotor Responses to CRD

Abdominal withdrawal reflex (AWR) scoring was conducted after the end of the intervention on the 36^th^ and 43^rd^ days. The AWR scoring standard was based on the experimental procedure of Al-Chaer et al. [15]. To reduce fecal formation before scoring, the rats were fasted but still had access to water for 8-12 h. The CRD stimulation method was as follows: a self-made balloon stimulator, a medical sphygmomanometer, and a syringe were connected by a 3-way valve; the balloon was inserted into the descending colon from the anus along the physiological curvature of the rectum in awake rats, and colonic stimulation via constant pressure dilatation was conducted under 4 different pressures of 20, 40 60, and 80 mm Hg. AWR scores were measured 3 times for each rat under each pressure. Each stimulation lasted approximately 20 seconds. The interval between the same pressure was 1 min, while the interval between different pressures was 4 min, and the mean value was taken as the final score. The standard for the AWR scoring was based on the AWR scoring system of Al-Chaer et al. [15].

### 2.4. Grouping and Intervention

The neonatal rats were randomly divided into normal group (NG) and model group (MG), rats in the NG did not receive any intervention, and the model rats were given CRD stimulation to establish the chronic visceral hypersensitivity rat model. After modeling, rats in the MG were randomly divided into MG, EA, P2X_3_ receptor antagonist (A-317491), and P2X_3_ receptor agonist (*α*, *β*-meATP) groups, 6 rats in each group. Rats in the NG and MG groups received the same fixation as the EA group. Rats in the EA group received EA intervention on bilateral ST25 and ST37 using the Han's meridian stimulator (2/100 Hz, 1 mA) for 30 min, and the depth of acupuncture was 5 mm, once a day for 7 days; rats in the A-317491 group received an intrathecal injection of A-317491 (10 *μ*l, 10 *μ*mol/*μ*l, Sigma) [16] every three days (day 1, day 3, day 7); rats in the *α*, *β*-meATP group received an intrathecal injection of *α*, *β*-meATP (10 *μ*l, 10 nmol/*μ*l, Sigma) [17] every three days as the A-317491 group.

### 2.5. Retrograde Tracing

The colonic primary sensory neurons were labeled in a retrograde manner 10 days before the electrophysiological experiment. The specific method was as follows: rats were anesthetized intraperitoneally with 2% pentobarbital sodium at a dose of 0.25 ml/100 g. The lower abdomen was shaved, and the colon was fully exposed in a sterile environment. A 32G needle connected to a Hamilton microsyringe (10 *μ*l) was used to slowly inject DiI (1 mM, Beyotime Biotechnology Co., Ltd., C1036) into the intestinal wall of the colon. After the injection was done, the needle remained still for 1 min and was then pulled out and wiped with a cotton ball to remove the leaked dye. Injections were conducted at 10 points, with 1 *μ*l per point. After the injection, the rat abdominal cavity was sutured, local disinfection was conducted using iodophor, and the rats were then kept in a single cage for recovery.

### 2.6. Hematoxylin-Eosin Staining

The colon tissues were fixed with 4% paraformaldehyde for 12 h before conventional dehydration and then cut into 4 *μ*m sections after paraffin embedding. Sections were deparaffinized by immersion in xylene (15 min ×2) and rehydrated using a graded ethanol series (100% for 5 min, 90% for 5 min, 80% for 5 min, and 70% for 5 min). Next, the sections were stained with hematoxylin solution for 1 min followed by 2 s in 1% acid ethanol and then rinsed in running water for 10 min. Then, the sections were stained with eosin solution for 5 min and dehydrated in graded alcohol (70% for 1 min, 80% for 1 min, 90% for 2 min, and 100% for 2 min) and cleared in xylene (15 min ×2). The mounted sections were then examined and photographed using an Olympus BX53 microscope.

### 2.7. Immunofluorescence

After 48 h of fixation with 4% paraformaldehyde, colon tissues were dehydrated overnight with 30% sucrose solution. Then, the mucosal layer, submucosal, circular muscle layer, serosal layer, and longitudinal muscle layer were successively removed under the magnifying microscope, and the intestinal plexuses were obtained. Intestinal plexuses that had been spread onto slides were incubated with 0.3% H_2_O_2_ for 20 min, followed by washing (5 min ×2) with distilled water. The slides were then blocked in normal goat serum at room temperature for 20 min. Next, drops of primary antibody were added, and the slides were incubated at 37°C for 2 h (anti-P2X_3_ primary antibody, Abcam, ab10269, 1 : 1000 dilution), followed by washing (5 min ×3) with 0.01 M PBS. The secondary antibody (goat anti-rabbit IgG-Cy3, BOSTER Biological Technology, BA1032, 1 : 50 dilution) was then added and incubated in dark for 30 min at room temperature, followed by washing (5 min ×3) with 0.01 M PBS and mounting in an antiquenching mounting solution. Image acquisition was conducted using an Olympus-BX53 microscope.

### 2.8. Immunochemistry

The DRG tissues were fixed with 4% paraformaldehyde for 12 h before conventional dehydration and then cut into 4 um sections after paraffin embedding. The sections were routinely deparaffinized, rehydrated, and incubated with 0.3% H_2_O_2_ for 20 min, followed by washing (5 min ×2) with distilled water. The slides were then blocked in normal goat serum at room temperature for 20 min. Next, drops of primary antibody were added, and the slides were incubated at 37°C for 2 h (anti-P2X_3_ primary antibody, Abcam, ab10269, 1 : 1000 dilution), followed by washing (5 min ×3) with 0.01 M PBS. The secondary antibody (goat anti-rabbit IgG, BOSTER Biological Technology, BA1003, 1 : 100 dilution) was then added and incubated at room temperature for 30 min, followed by washing (5 min ×3) with 0.01 M PBS and staining with diamino-benzidine (DAB) and hematoxylin. Image acquisition was conducted using an Olympus-BX53 microscope, and Image-Pro PLUS software was used for the data acquisition.

### 2.9. Real-Time Polymerase Chain Reaction

To extract total RNA from tissues, the tissue was homogenized, and total RNA was extracted using the TRIzol method, followed by the determination of concentration, purity, and integrity. An equal amount of total RNA was taken to synthesize complementary DNA (cDNA) via reverse transcription using a SYBRGreen PCR kit (QIAGEN 208052) and a cDNA synthesis kit (Invitrogen K1622). The sequences of the primers used were as follows (the primers were designed by ABI's Prime Express Software v2.0 and synthesized by Beijing Genomics Institute): P2X3-F: ACCCCACCCCAGAATGAAGA, P2X3-R: AGCTGTAGTTCACGCAGCGG; and rGAPDH-F: GGCAAGTTCAACGGCACAGT, rGAPDH-R: ATGACATACTCAGCACCGGC. The data were analyzed using the ABI Prism 7500 SDS Software embedded in the instrument.

### 2.10. Dissociation of DRG Neurons

The previously extracted DRG were placed in oxygen-permeable extracellular fluid at 4°C. The outer membrane covering the surface was peeled off with the aid of a dissection microscope, and the sample was then digested in 1 ml of digestion solution containing 1 mg/ml trypsin (Gibco, 25200-056), 1.5 mg/ml type II collagenase (Worthington, LS004176), and 6 mg/ml bovine serum albumin (Beyotime, ST023) for 45 min at 37°C. After digestion, the mixture was gently pipetted to generate a single-cell suspension and centrifuged (300 g, 2 min, 4°C). The supernatant was discarded, and the pellet was resuspended with an appropriate amount of oxygen-saturated extracellular fluid and finally placed onto slides. The cells were allowed to adhere to the slides, which were used for patch clamp recording after 1.5 h.

### 2.11. Patch Clamp Recording

The prepared slides with cells were placed in oxygen-saturated extracellular fluid (150 mM NaCl, 5 mM KCl, 2.5 mM CaCl_2_, 1 mM MgCl_2_.6H_2_O; 10 mM glucose; 10 mM HEPES, pH adjusted to 7.4 with NaOH). Fluorescently labeled cells were selected as experimental subjects with a fluorescence microscope. The pipette was pulled with a P-1000 pipette pulling device. The tip of the pipette was 1 to 2 *μ*m in diameter and filled with the pipette solution (140 mM KCl, 1 mM MgCl_2_.6H_2_O, 5 mM EGTA, 3 mM Na_2_ATP, 0.2 mM Na_3_GTP, 10 mM HEPES, pH adjusted to 7.2 with KOH), and the impedance was 3 to 5 M*Ω*. The recording frequency was 1 kHz, the acquisition frequency was 10 kHz, and the clamping voltage was -70 mV. In the current clamp mode, a current stimulus with an intensity of -200 to 340 pA and a duration of 800 ms was applied, and the stimulation frequency was 0.0033 Hz, that is, one current stimulus every 3 sec. The minimum stimulation current that caused the cell to generate an action potential (AP) was used as the rheobase, and the resting membrane potential (RMP) value, the rheobase value, and the AP frequency were recorded.

### 2.12. Analysis and Statistics

Statistical analysis was performed by SPSS 21.0 software. If the data followed a normal distribution, the data were expressed as $\bar{x}\pm s$, and if not, the data were expressed as M (QU-QL). When analyzing the data, If the data were normally distributed and the variances were homogeneous, One-way ANOVA was used, and the least significant difference (LSD) *t*-test was used for pair-wise comparisons. If the data followed a normal distribution but the variances were not homogeneous, One-way ANOVA was used for analysis, and the Games-Howell method was used for pair-wise comparisons. If the data did not follow a normal distribution and the variances were not homogeneous, then the Kruskal-Wallis H test was used for analysis, and the Nemenyi method was used for pair-wise comparisons. The test level was *α* = 0.05, and *P* < 0.05 determined statistical significance.

## 3. Results

### 3.1. Microscopic Assessment of the Colon

**Figure 1:**
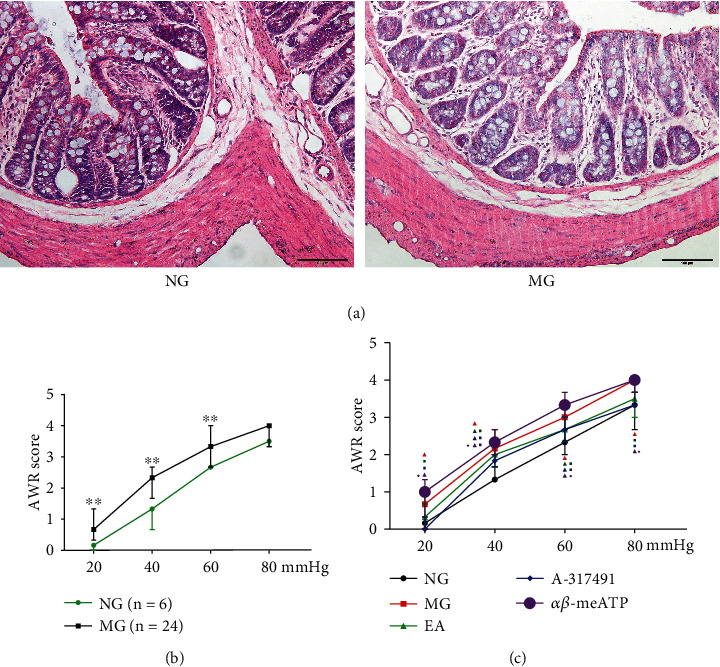
HE staining of colon tissue and AWR scores. (a) HE staining of colon tissue (scale bar = 100 *μ*m); (b) Behavioral scores before EA and drug intervention (NG: *n* = 6; MG: *n* = 24). ^∗∗^*P* < 0.01 vs. NG. (c) Behavioral scores after EA and drug intervention (*n* = 6). ^▲^*P* < 0.05 vs. NG; ^▪^*P* < 0.05 vs. MG; ^♦^*P* < 0.05 vs. EA.

Local inflammation was not observed in the IBS chronic visceral pain animal model induced by mechanical CRD in neonatal rats, and the pathological manifestations in the colon were mainly behavioral. The staining for pathology of the colon (HE staining) revealed that the colonic mucosa was intact in the visceral pain rats, and the gland bodies were neatly arranged, with a small amount of eosinophilic infiltration in the lamina propria and no interstitial edema, showing no significant difference with the normal rats (Figure 1(a)), consistent with the clinical colon pathology of IBS.

### 3.2. EA Relieves Visceral Hypersensitivity

We firstly observed changes in the behavior of rats. In the 6th week, the behaviors of the rats under different CRD intensities (20 mm Hg, 40 mm Hg, and 60 mm Hg) were evaluated. The model group showed increased colonic sensitivity (*P* < 0.01, vs. NG, Figure 1(b)). Under stimulation with the CRD intensity of 80 mm Hg, there was no significant difference between the MG and NG (*P* > 0.05, Figure 1(b)), but the AWR score of the rats in the MG was increased. Therefore, from the perspective of behavior, the IBS visceral pain hypersensitive rat model was successfully produced. The visceral pain rats included in the experiment were then randomly divided into an MG, EA, A-317491, and *α*, *β*-meATP groups. The AWR scores after EA treatment showed that the scores of MG were significantly increased at each pressure level (*P* < 0.01, vs. NG, Figure 1(c)), confirming the persistence of visceral hypersensitivity after CRD stimulation. The scores of EA and A-317491 groups decreased at each pressure level (*P* < 0.05, vs. MG, Figure 1(c)), indicating that EA can reduce visceral hypersensitivity in the model rats and that of A-317491 reduced visceral hypersensitivity by blocking peripheral P2X_3_ receptors, whereas *α*, *β*-meATP increased visceral hypersensitivity by activating P2X_3_ receptors (Figure 1(c)). EA can improve the visceral hypersensitivity of the IBS rats and increased the pain threshold.

### 3.3. P2X_3_ Protein and mRNA Expression in the Myenteric Plexus

**Figure 2:**
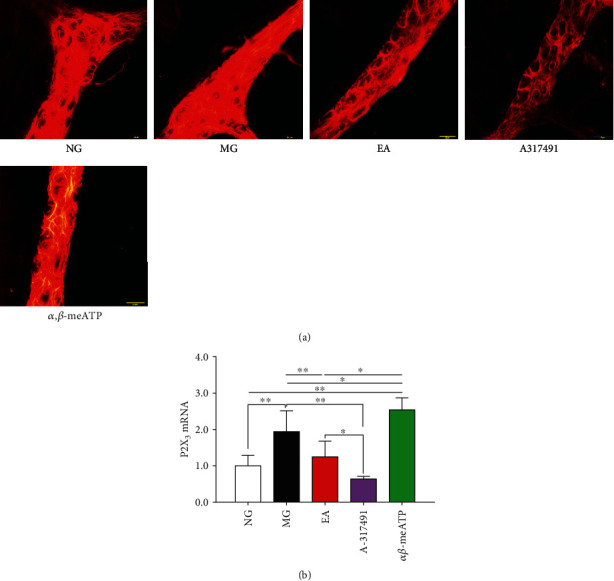
P2X_3_ protein and mRNA expression in the colonic myenteric plexus. **(**a) Distribution of the P2X_3_ receptor in the colonic myenteric plexus (scale bar =20 *μ*m). (b) P2X_3_ mRNA expression in colon. ^∗^*P* < 0.05, ^∗∗^*P* < 0.01.

The P2X_3_ receptor was expressed in the colonic myenteric plexus of the rats in each group, mainly in the intermuscular neurons of the colon. CRD stimulation in neonatal rats upregulated the expression of P2X_3_ receptor in the colonic myenteric plexus (Figure 2(a)). The *α*, *β*-meATP could further upregulate the expression level (Figure 2(a)), while A-317491 and EA could downregulate the expression level (Figure 2(a)). Subsequently, we observed this regulatory effect at the mRNA level of the P2X_3_ receptor. CRD stimulation in neonatal rats induced an increase in P2X_3_ mRNA expression in the colon tissue (*P* < 0.01, MG vs. NG, Figure 2(b)). The *α*, *β*-meATP further promoted an increase in the expression level (*P* < 0.05, vs. MG, Figure 2(b)), while A-317491 and EA inhibited P2X_3_ mRNA expression (*P* < 0.01, vs. MG, Figure 2(b)), consistent with the previous results, suggesting that EA may relieve the visceral hypersensitivity by directly regulating the P2X_3_ mRNA expression in the colon.

### 3.4. P2X_3_ Expression in DRG Neurons

**Figure 3:**
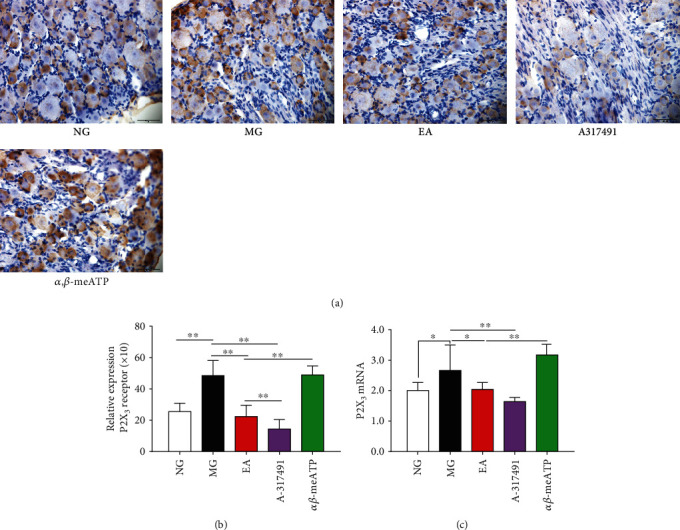
P2X_3_ receptor protein and mRNA expression in colon-associated DRG. **(**a) Immunohistochemical detection of the distribution of P2X_3_ receptor positivity (*n* = 6, scale bar = 50 *μ*m). (b). Semiquantitative analysis of the P2X_3_ receptor protein in DRG (*n* = 6). (c). Relative expression of P2X_3_ mRNA in DRG (*n* = 6). ^∗^*P* < 0.05, ^∗∗^*P* < 0.01.

The P2X_3_ receptors are most highly expressed in small-diameter neurons of the DRG [18–20]. The distribution of P2X_3_ receptor was detected by positive results in an immunohistochemistry analysis. The results of the semiquantitative analysis showed that P2X_3_ was widely distributed in small-diameter DRG neurons, and the staining was light (Figure 3(a)). CRD stimulation in neonatal rats upregulated the P2X_3_ receptor protein expression, and the staining was darker, with positive or strongly positive reactivity (*P* < 0.01, vs. NG, Figure 3(b)). The EA and A-317491 exhibited an inhibition of the CRD-induced abnormally upregulated P2X_3_ expression (*P* < 0.01, vs. MG, Figure 3(b)), while *α*, *β*-meATP upregulated P2X_3_ expression (Figure 3(b)), consistent with the increased abdominal pain behavioral scores induced by CRD stimulation (Figure 1(c)). A real-time PCR assay was used to detect the P2X_3_ mRNA expression in DRG. CRD stimulation affected the expression of the P2X_3_ receptor at the mRNA level, with a significant upregulatory effect (*P* < 0.05, vs NG, Figure 3(c)). The *α*, *β*-meATP increased P2X_3_ mRNA expression (Figure 3(c)), while A-317491 and EA downregulated P2X_3_ mRNA expression (*P* < 0.05, vs. MG, Figure 3(c)), consistent with the protein detection results, indicating that the regulation of P2X_3_ by EA may occur at the transcription stage.

### 3.5. EA Regulates Electrophysiological Properties of Colon-Associated DRG Neurons

**Figure 4:**
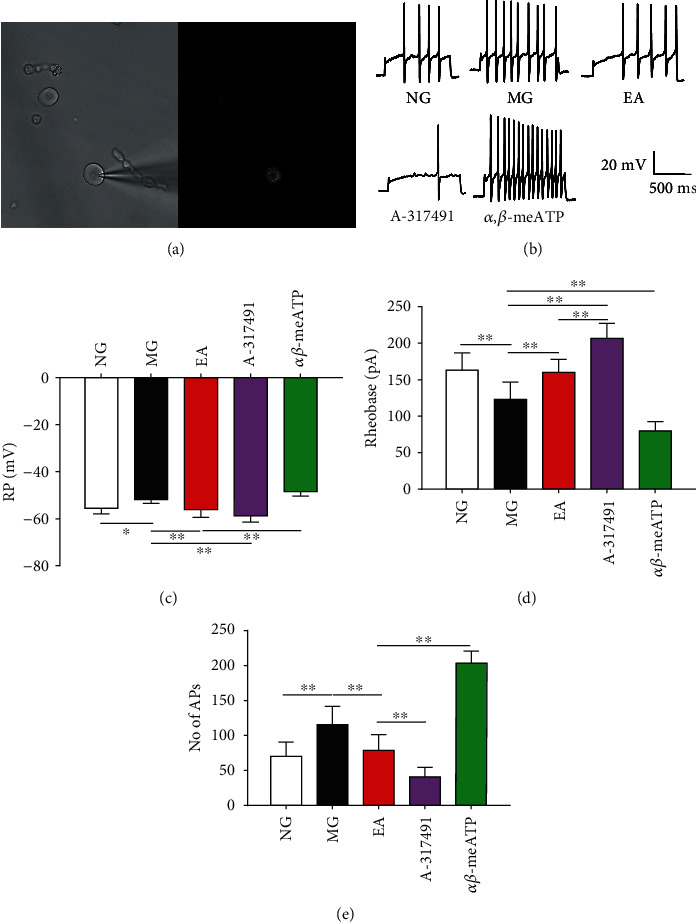
Changes in the membrane electrical properties of the colon-associated DRG neurons. (a) The left figure shows differential interference contrast (DIC) imaging of DRG neurons after digestion, and the right figure shows fluorescence imaging of colon-associated neurons labeled with DiI. (b) Representative traces of APs after a 200 pA depolarization current injection into DiI-labeled neurons. (c) Changes in the cell RMP in colon-related DRG neurons. (d) Changes in the rheobase in colon-related DRG neurons. (e) Number of APs of neuronal cells in colon-related DRG. ^∗^*P* < 0.05, ^∗∗^*P* < 0.01.

It has been reported that the excitability of colon-associated DRG neurons in rats with IBS visceral hyperalgesia is increased [21, 22], which can persist into adulthood. In this study, we observed whether EA has a regulatory effect on this elevated neuronal excitability. In our previous molecular biology experiments, we found that EA can inhibit the expression of P2X_3_ receptor in the P2X_3_-positive neurons in DRG related to nociceptive information. This study aimed to use electrophysiological methods to further explore whether P2X_3_ receptor is involved in the inhibition of the peripheral sensitization to visceral pain by EA. The excitability of DRG neurons was significantly increased by CRD stimulation in neonatal rats, consistent with the behavioral experiment (Figure 1(c)). Colonic sensory neurons were labeled in a retrograde manner (Figure 4(a)), and the cells on the right side with DiI-positive labeling were projection neurons of colon. We used a whole-cell recording method to record changes in the excitability of this type of neuron.  Figure 4(b) depicts a representative trace of the AP propagation. The results showed that CRD stimulation in neonatal rats significantly upregulated the RMP of colon-associated DRG neurons (*P* < 0.05, vs. NG, Figure 4(c)) and downregulated the rheobase intensity of colonic neurons (the minimum current value required to induce an AP, *P* < 0.01, vs NG, Figure 4(d)), which also induced an increase in the number of APs propagated by rat colonic neurons (the number of APs with a stimulation of 200-340 pA current intensity) (*P* < 0.01, vs. NG, Figure 4(e)). We used EA (2/100 Hz, 1 mA, 30 min) as an intervention in rats with IBS visceral hypersensitivity and found that EA significantly inhibited the excitability of colonic sensory neurons in the IBS rats, including lowering the RMP (*P* < 0.01, vs. MG, Figure 4(c)) and upregulating the rheobase of colonic neurons (*P* < 0.01, vs. MG, Figure 4(d)). EA also inhibited the propagation of APs in sensitized colonic neurons (*P* < 0.01, vs. MG, Figure 4(e)), consistent with previous studies [22, 23]. We used intrathecally injected drugs to selectively antagonism the P2X_3_ receptor in DRG neurons. A-317491 regulated the membrane electrical properties of the colonic neurons, including the RMP, rheobase, and APs (*P* < 0.05, vs. MG, Figure 4(c)–4(e)). Combined with the previous behavioral and molecular biological results, we speculate that P2X_3_ receptors in the primary afferent neurons play an important role in mediating the alleviation of visceral pain by EA.

## 4. Discussion

The P2X_3_ receptor in colon-associated DRG neurons plays an important role in ATP-mediated pain in IBS rats with visceral hypersensitivity [24]. Burnstock found that upon distension stimulation, the epithelial cells of tubular organs and cystic organs in the body (such as the intestine, ureter, and bladder) release ATP, which acts on the P2X_3_ and P2X_2/3_ receptors of afferent nerve endings under the endothelium, triggering the transmission of pain signals to the central nervous system [8, 25] and thereby participating in a wide range of biological effects, including visceral pain signal transduction [8, 25, 26]. The P2X_3_ receptor is highly selectively expressed in the medium- and small-diameter DRG neurons in peripheral primary sensory afferent nerves and plays an important role in the transmission, development, and maintenance of pain signaling and pain sensitization [27, 28]. We have previously reported on the regulation by EA of the central sensitization to visceral pain and found that EA has different degrees of regulation on P2X_3_-mediated central sensitization in the anterior cingulate cortex (ACC) and prefrontal cortex (PFC) [16], as well as the ventral posterolateral nucleus (VPL) (unpublished). We also found that the P2X_3_ receptor also plays an important role in the peripheral sensitization to visceral pain. While the above studies lacked the support of electrophysiological evidence.

This study mainly used electrophysiological recording methods to investigate the mechanism of acupuncture in the regulation of the peripheral sensitization to visceral pain. The results of the molecular biotechnology tests are consistent with our previous reports [16, 29, 30]. The behavioral AWR scores showed that EA (2/100 Hz, 1 mA, 30 min) significantly alleviated the visceral hypersensitivity of IBS. The intrathecal injection of the P2X_3_-selective antagonist A-317491 significantly reduced the AWR score, proving from the behavioral perspective that P2X_3_ plays an important role in visceral hypersensitivity. We detected the P2X_3_ receptor protein and mRNA expression levels in DRG neurons and found that EA could inhibit the protein and mRNA expression of P2X_3_ receptor in IBS rats. To continue to explore the electrophysiological mechanism of the P2X_3_ receptor of DRG neurons in the EA-mediated alleviation of visceral pain, we performed interventions focusing on the P2X3 receptor at overall level and observed the effect of EA on the membrane electrical properties of colon-associated DRG neurons. We observed the effects of EA on the RMP, rheobase, and AP of neurons and found that EA significantly downregulated the RMP of colonic sensory neurons (with positive DiI labeling), upregulated the rheobase of sensitized neurons, and inhibited the number of APs propagated of sensitized neurons. This result indicates that EA can significantly inhibit the excitability of peripheral neurons and reduce the peripheral sensitization status, consistent with previous studies [23].

Based on the effects of P2X_3_ receptor on the colon-associated neurons, we examined the P2X_3_ receptor protein and mRNA expression levels in the sensory nerve peripheral end (sensory periphery) in colon and found that the expression of P2X_3_ receptor was upregulated. EA can partially inhibit this upregulation, indicating that EA can regulate the activity of P2X_3_ at the peripheral end of the intestinal sensory nerve, which is the visceral pain effector. At the central region of the sensory nerve (the superficial layer of the spinal dorsal horn), our previous studies found that EA can also inhibit the CRD-induced upregulation of P2X_3_ receptor expression in the lamina I and II of the spinal dorsal horn [16]. The above experiments provide experimental evidence for P2X3 receptor of sensory neurons participating in electroacupuncture to relieve chronic visceral hypersensitivity in IBS rats.

While acupuncture is known to regulate the environment for processing pain information in the body through multiple systems, multiple levels, and multiple targets [31–33]. There are still some unresolved problems in this study that require further research. For example, the detailed molecular mechanisms by which the mechanical stimulation of EA regulates P2X_3_ receptor-related neuronal excitability are still unclear. We recently found that EA has a regulatory effect on the surface P2Y_1_ receptor in the colon-associated neurons in the DRG (unpublished). It is known that P2X_7_ on the surface of satellite glial cells can upregulate the activity of P2Y_1_ in neurons and thus inhibit the activity of the nociceptive receptor P2X_3_ [34–36], and EA has a significant inhibitory effect on satellite glial activity around colon-associated neurons (unpublished). We speculate that EA may play a role via the inhibitory pathway of P2X_7_-P2Y_1_-P2X_3_ in the “neuron-glial” information interaction. However, more experimental evidence is needed to support this hypothesis.

## 5. Conclusions

In conclusion, CRD stimulation in neonatal rats upregulated the protein and mRNA expression of P2X_3_ receptor in the colon and colon-associated DRG neurons, and increased the excitability of colon-associated DRG neurons. EA downregulated the increased P2X_3_ receptor expression in the colon and colon-associated DRG neurons and inhibited the excitability of colon-associated DRG neurons induced by CRD stimulation in neonatal rats, consistent with P2X_3_ receptor selective antagonist A-317491. Combined with the previous researches, we speculate that the regulation of P2X_3_ receptors in the primary afferent neurons may be the important neurobiological mechanism by which EA relieves visceral hypersensitivity in IBS rats.

## Data Availability

The initial data used to support the findings of this study are included within the article.
